# Reliability and applicability of the Patient Enablement Instrument (PEI) in a Swedish general practice setting

**DOI:** 10.1186/s12875-015-0242-9

**Published:** 2015-03-04

**Authors:** Mattias Rööst, Andrzej Zielinski, Christer Petersson, Eva Lena Strandberg

**Affiliations:** Department of Research and Development, Region Kronoberg, Box 1223, SE-351 12 Växjö, Kronoberg Sweden; Department of Clinical Sciences, General Practice/Family Medicine, Lund University, Malmö, Sweden; Blekinge Centre of Competence, Blekinge County Council, Karlskrona, Sweden

**Keywords:** Consultation, Enabling factors, Patient-centred care, Primary care, Patient evaluation

## Abstract

**Background:**

The Patient Enablement Instrument (PEI), which has been used to evaluate core ingredients in primary care consultations, has been proposed as a means of moving beyond patient satisfaction evaluations. The aim of the present study was to examine the reliability and applicability of the PEI to the Swedish context.

**Methods:**

The original PEI was translated to Swedish and included in a questionnaire that was given to consecutively scheduled patients in four primary care settings. Respondents completed identical questionnaires immediately after a consultation, as well as two days and two weeks later. The analysis focused on internal reliability, test-retest reliability and internal construct validity.

**Results:**

Mean PEI scores declined significantly between baseline (3.48, SD 3.21) and the first follow-up questionnaire (3.06, SD 3.37). All three questionnaires showed high internal consistency (Cronbach’s alpha >0.85). Test-retest showed moderate agreement for all questions when comparing baseline and the first follow-up (kappa 0.54-0.65) and greater consistency between the two follow-up questionnaires (kappa 0.65-0.75). A large proportion of respondents characterized at least one of the questions as irrelevant (39%).

**Conclusions:**

The Swedish version of the PEI instrument has high internal consistency and moderate to good reliability. It can be used in research but is not recommended as a measure of quality of care. The instrument could benefit from further development and validity testing.

**Electronic supplementary material:**

The online version of this article (doi:10.1186/s12875-015-0242-9) contains supplementary material, which is available to authorized users.

## Background

Various aspects of patient-doctor consultations have been examined by several studies with the aim of defining good quality of care in general practice (GP). However, given that GP consultations often deal with multi-dimensional problems, single-objective measures have limited value. As a result, the focus has been on methods of evaluating perceived core ingredients of a consultation, such as patient-centeredness, empowerment, and holism. Patient satisfaction has been a traditional outcome measure in trying to capture such dimensions, but the argument has been made that the measure is related more to patient expectations than to actual outcomes [1]. The Patient Enablement Instrument (PEI) has been suggested as a means of examining various aspects of a clinical consultation for the purpose of capturing dimensions other than patient satisfaction [2]. The instrument focuses instead on the impact of a consultation on a patient’s self-perceived ability to understand and cope with health issues and disease. The original PEI version was developed by Howie et al. from literature reviews and patient interviews to evaluate enablement after a clinical consultation in primary health care. The instrument has been described to be related to, but different from, measures of satisfaction [2]. The original PEI has been translated and evaluated in several countries, generally exhibiting high internal reliability [3-6]. Factors associated with higher enablement scores include continuity and longer consultations [2,6], the patient’s rating of the doctor’s communication skills [7], perceived empathy in the doctor [8], and the patient’s ethnic minority status [2,7,9]. Age and health needs, as well as other patient-centred variables, have shown inconsistent correlation with PEI values [7,10,11]. However, studies concerning the relevance and applicability of the various questions are lacking. Apart from being used as a research instrument it has been suggested that PEI could be included in a score system of individual doctors’ ability for patient-centeredness in the clinical situation and linking this to financial incentives [12]. As that could have a large impact on primary health care providers it is of major importance that such instrument meet high quality levels and is proven robust and applicable.

There are no published studies of patient enablement in Sweden. An unpublished pilot study in 2000 generated relatively high PEI scores but concluded that the instrument might not be applicable in the Swedish context, partly due to major differences among doctors and the absence of confirmation of discrepancies in PEI scores related to the length of the consultation, as had been shown in previous studies (Andersson SO. Patient Enablement Instrument. Erfarenheter från en pilotstudie i Sverige (Patient Enablement Instrument. Experiences from a pilot study in Sweden). Unpublished). The study did not further investigate the internal dimensions of the instrument.

The aim of the present study was to investigate the applicability of the PEI in the Swedish context, including determination of reliability in a general primary health care population.

## Methods

### Setting

This was a cross-sectional study of the PEI, covering appointments at four primary health care centres in the Swedish counties of Kronoberg and Blekinge. Primary practice in Sweden differs from that in other European countries by generally longer consultations, around 30 minutes, often attending several health problems during one meeting. Acutely ill patients generally receive appointments on the same day at these centres, while check-ups for chronic and long-term symptoms are scheduled in advance. Only check-ups were included in this study.

### Study population and data collection

Consecutively scheduled patients age 18 and older were asked by the clinic’s reception staff to participate in the study once they had received verbal and written information. Because the study sought to evaluate the instrument in a general primary care population, there were no other exclusion criteria. Patients received three identical questionnaires concerning age, gender, and the Swedish version of the PEI instrument. They were asked to complete the first questionnaire (Q1) immediately after their consultation and leave it at the reception desk. They were also asked to complete and return identical follow-up questionnaires in pre-addressed envelopes two days (Q2) and two weeks (Q3) later. As respondents remained completely anonymous during the data collection process reminders could not be sent out. Data collection lasted from April to August 2013.

### The instrument

The original PEI consists of six questions intended to reflect patient enablement. The questions are structured according to the following. “As a result of your visit to the doctor today, do you feel you are a) able to cope with life, b) able to understand your illness, c) able to cope with your illness, d) confident about your health, e) able to help yourself?” The questions focus on understanding and coping with health issues and illness as the result of a consultation. The answers are graded on a three-point scale – “same or less” or “not applicable” (0), “better/more” (1), and “much better/much more” (2). Thus, the total PEI score ranges between 0 and 12 [2].

The study involved a two-step translation process to create a Swedish version of the instrument (see Additional file 1). First, two members of the research team translated the original English version of the PEI instrument to Swedish, paying special attention to the meaning of the questions. Second, a native English speaker, who was blinded to the original PEI, translated the instrument back to English. The translated and back-translated versions were carefully reviewed by the research team, and the few discrepancies required some minor changes to the Swedish version while preserving the original meaning of all six questions. This only included exchanging two words with synonyms in Swedish.

### Data analysis

Descriptive statistics was used to determine mean values and standard deviations (SD) of PEI scores in each of the three questionnaires, as well as stratified by gender and age (<65/65 and older). Since the data were non-normally distributed, also median and interquartile range (IQR) was presented. Mann–Whitney U tests were used to compare total scores stratified by gender and age. Wilcoxon signed rank test was used to compare PEI scores between questionnaires. Because there is no gold standard with which to compare results, special attention was paid to analysing “not applicable” responses on the hypothesis that they reflected perceived relevance. Descriptive statistics were used to identify the individual questions and patterns of questions that were most commonly characterised as not applicable in the three questionnaires. The significance of the differences was analysed by a chi 2 test.

The internal reliability of each of the three questionnaires was measured by Cronbach’s alpha coefficient presented with 95% confidence intervals (95%CIs), with a value of >0.7 defined as acceptable [13]. Test-retest reliability was determined by (a) Wilcoxon signed rank test comparing total mean scores between Q1 and Q2, as well as between Q2 and Q3, (b) Cronbach’s alpha coefficient to determine the consistency of each question among the three questionnaires, (c) Kappa statistics to compare the responses to each question between Q1 and Q2, as well as between Q2 and Q3. Spearman’s correlation was employed to analyse the internal construct validity of each questionnaire by determining the correlation between each question and the total PEI score. All data analyses were performed by SPSS 20.

### Ethical considerations

Under Swedish law, ethical approval was not required for this study since it was a quality improvement project. All respondents were informed that participation was voluntary and anonymous.

## Results

Questionnaires were given to 167 patients. The majority of patients (n = 153, 92%) responded to at least one of the questionnaires. The response rate declined with each succeeding questionnaire (Q1 = 148, Q2 = 86, Q3 = 72), and only 68 patients (41%) responded to all three of them. The mean age of respondents to Q1 was 56.5, SD 17.3 (95%CI 53.7-59.3), and 54% of them were women.

Table 1 shows the number of respondents and total PEI scores for the three questionnaires. The mean score was 3.48 (SD 3.21) for Q1, 3.06 (SD 3.37) for Q2 and 3.31 (SD 3.52) for Q3. Median score was 3 (IQR 0.25-6) in Q1, 2 (IQR 0–6) in Q2 and 2.5 (IQR 0–6) in Q3. A Wilcoxon signed rank test showed that PEI scores were significantly higher on Q1 than Q2 (Z = −2.90. p = 0.01). There was no significant difference between Q2 and Q3 (Z = −0.39, p = 0.70). Mann–Whitney U tests showed no significant differences in PEI scores in Q1 related to gender (U = 2462, Z = −1.00, p = 0.32) or age (U = 2186, Z = −1.74, p = 0.08).

**Table 1:** **Respondents and mean total PEI score for the three questionnaires**

	**Q1 (n)**	**Mean PEI Q1 (SD)**	**Q2 (n)**	**Mean PEIQ2 (SD)**	**Q3 (n)**	**Mean PEI Q3 (SD)**
Total	148	3.48 (3.21)	86	3.06 (3.37)	72	3.31 (3.52)
Women	80	3.29 (3.25)	48	3.08 (3.32)	42	2.86 (3.13)
Men	68	3.71 (3.16)	38	3.03 (3.48)	30	3.93 (3.98)
Age <65	89	3.13 (3.16)	48	2.73 (3.31)	39	2.90 (3.31)
Age 65-	59	4.00 (3.24)	38	3.47 (3.42)	33	3.79 (3.76)

Table 2 describes the frequency of the “not relevant” response for each question. A large proportion of respondents characterised at least one of the questions on Q1 as not relevant (39%). Four per cent responded that all of the questions were not relevant. The frequency of “not relevant” responses declined for Q2 and Q3. The question most commonly characterised as not relevant was “able to keep yourself healthy” (25.3%), followed by “able to cope with life” (22.5%). There was a high correlation of “not relevant” responses between these two questions. When all variables in Q1 were included, “not relevant” responses were significantly more common to “able to keep yourself healthy” than to the other questions (*χ*^2^(5, N = 901) = 17.02, p = 0.004). When the above question was excluded, “not relevant” responses were more common to “able to cope with life” than to other questions at almost a significant level (*χ*^2^(4, N = 751) = 9.39, p = 0.05). In Q2 and Q3, no significant differences could be determined.

**Table 2:** **Number of respondents (valid percentage) who said that the questions were not relevant on the baseline questionnaire (Q1), follow-up after two days (Q2), and follow-up after two weeks (Q3)**

	**Q1**	**Q2**	**Q3**
able to cope with life	34 (22.5)	14 (16.3)	10 (13.9)
able to understand your illness	23 (15.3)	10 (11.6)	5 (6.9)
able to cope with your illness	24 (16.0)	8 (9.3)	7 (9.7)
able to keep yourself healthy	38 (25.3)	13 (15.1)	11 (15.3)
confident about your health	15 (10.0)	7 (8.1)	4 (5.6)
able to help yourself	21 (14.0)	10 (11.6)	5 (6.9)
all questions	6 (4.0)	3 (3.5)	3 (4.2)

Cronbach’s alpha coefficient of internal reliability was higher than 0.85 for all three questionnaires (0.86, 95%CI 0.82-0.89 for Q1, 0.88, 95%CI 0.83-0.91 for Q2, and 0.90, 95%CI 0.87-0.94 for Q3).

Test-retest using kappa statistics showed moderate agreement for all questions when comparing baseline and the first follow-up (kappa values of 0.54-0.65) and greater consistency between the first and second follow-ups (kappa values of 0.65-0.75) (Table 3). Most questions showed a correlation of >0.7 with the total PEI score (Table 4). The questions “able to cope with life” and “able to keep yourself healthy” showed lower levels of correlation (Spearman <0.7).

**Table 3:** **Correlation between individual questions on the three questionnaires (Kappa)**

	**Inter-questionnaire correlation**	
	Q1vsQ2	Q2vsQ3
cope with life	0.54	0.74
understand your illness	0.65	0.75
cope with your illness	0.61	0.65
keep yourself healthy	0.56	0.61
confident about your health	0.54	0.74
able to help yourself	0.57	0.65

Q1vsQ2: Correlation between responses to individual questions on questionnaire 1 (Q1) and questionnaire 2 (Q2).

Q2vsQ3: Correlation between responses to individual questions on questionnaire 2 (Q2) and questionnaire 3 (Q3).

**Table 4:** **Correlation between individual questions and the total score (Spearman)**

	**Intra-questionnaire correlation**		
	**Q1**	**Q2**	**Q3**
cope with life	0.63	0.67	0.71
understand your illness	0.73	0.74	0.82
cope with your illness	0.77	0.76	0.78
keep yourself healthy	0.60	0.63	0.70
confident about your health	0.73	0.75	0.75
able to help yourself	0.74	0.70	0.85

## Discussion

The present study finds high internal consistency of the Swedish PEI version at both baseline and follow-up. There was a clear tendency, both at the total and single-item level, for scores to decline between baseline and the first follow-up and to be more consistent between the first and second follow-ups. Two questions were characterised as less relevant, and they also deviated more from the total score than the other questions.

The adequacy of using the PEI as a quality measure has been a matter of discussion [7,13]. The present study shows high internal consistency for all three questionnaires, as was the case for previous versions of the PEI [2,3,5]. Cronbach’s alpha of slightly below 0.9 indicates that the Swedish version can be used for research purposes but is not appropriate for individual clinical use, where 0.95 is desirable [14]. Thus, we do not believe that the instrument should be used to compare quality of care between clinics or individual doctors but is entirely suitable for researching factors related to enablement at the group level. The variability of the PEI due to several factors not related to the care provider, as well as a low predictive value for variances in the PEI due to known related factors [7,15] as found by other studies, also makes the instrument inappropriate as a general quality of care indicator.

For all individual questions, the correlation was lower between the original questionnaire and the first follow-up than between the first and second follow-ups. Test-retest also indicated that mean PEI scores declined during the first two days. A previous study of a French version of the PEI found a decline in mean PEI scores over two weeks and hypothesised that the observed difference was related to an actual change in patient enablement rather than a matter of reliability of the instrument [3]. Our findings support the hypothesis that enablement is highest immediately after the consultation and suggest subsequent stability at a lower level. Future studies should examine the degree of such stability over time. The results of our study indicate that the reliability of the instrument can be examined most effectively by comparing the two follow-up questionnaires.

Although enablement and the PEI have been widely studied, both theoretical and methodological questions remain. A large proportion (39%) of the respondents in our study characterised at least one of the PEI questions as not relevant, raising issues about the applicability of the instrument to the general population. The two questions most frequently characterised as not relevant (“able to cope with life” and “able to keep yourself healthy”) also deviated more from the total score than the other questions, indicating that they reflect separate considerations and that the instrument could benefit from further development. A large UK study with data that had been routinely collected from approximately 190,000 consultations excluded 16% from the analysis because more than one question had been characterised as not applicable [7]. The study only included three of the six PEI questions (“able to understand illness”, “cope with illness”, and “keep yourself healthy”). An issue for future studies to address is why a large percentage of respondents perceive the questions as not relevant or applicable to their situation. One reason may be that the questions have not been adapted to the nature and duration of the disease; the PEI might be more suitable for specific patient populations that are more amenable to the broad scope of the questions. For example, consultation and establishment of a patient-doctor relation might have a larger impact for understanding and coping with disease if the patient has a newly diagnosed chronic disease than if the patient has had the disease for a long time. Similarly, it is reasonable to believe that severity and physical as well as psychological effects of the disease impacts on the perceived applicability of the questions.

Although several studies have been conducted about the PEI and its determinants, questions remain about the definition and predictors of enablement. PEI scores have been tied to situational, doctor-related, and patient-related factors. Core components of general practice and patient-centred care, such as the patient’s perception of continuity and the doctor’s communication skills, have been related to PEI scores. However, an analysis that included known factors related to the PEI could explain only 16% of the variance in scores [7]. In other words, patient enablement is largely predicted by unknown factors.

Furthermore, the actual distinction between patient enablement and patient satisfaction has yet to be established. Despite the lack of empirical evidence, patient enablement has been theoretically linked to self-efficacy as reflected by that people are able to take certain health measures of their own. Satisfaction is generally understood as an outcome, while self-efficacy and enablement may be more predictive of future behaviour [10]. Other than a study of asthma patients [16], few studies have focused on the ability of the PEI to influence future health-related behaviours or outcomes. That study associated higher PEI scores with clinical improvement as measured by asthma quality of life questionnaires. To further validate the PEI and conceptualize enablement, we suggest studies about the clinical or behavioural effects of various PEI scores and that an effort be made to compare PEI scores with various measures while taking into account the situation before and after the consultation.

### Strengths and limitations

The major strength of the present study is the generalizability of the results as the Swedish version of the PEI was tested in a general patient population, the group for which it was designed [2]. However, our inclusion of unselected scheduled patients without further specifying the factors related to the individual, doctor or disease affects interpretation of the data. Comparability of our results to those presented by other studies is therefore limited to the dimensions of the instrument. As a consequence, due to the lack of context factors, exact PEI scores should not be compared with the results of previous studies. We used an anonymous data collection process to reduce the risk of influencing the responses. This strategy precluded sending reminders to non-responders and resulted in the study limitation that a fairly large quantity of data was lost in the follow-up questionnaires. The lower frequency of “not relevant” responses to the follow-up questionnaires is probably a result of greater homogeneity among the respondents, as those responding “not relevant” in the first questionnaire were less likely to provide follow-up data. Thus, we decided to focus on the results of the baseline questionnaire when analysing the internal consistency and applicability of the instrument.

## Conclusions

The Swedish version of the PEI instrument has high internal consistency and moderate to good reliability. The instrument can be applied in research but is not recommended as a measure of quality of care. The instrument could benefit from further development and validity testing.

## Additional file

Additional file 1:
**Swedish version of the PEI adapted from the original English version.**

## Abbreviations

CI: Confidence interval
GP: General practice
IQR: Interquartile range
PEI: Patient enablement instrument
SD: Standard deviation

## References

[1] Sitzia J, Wood N (1997). Patient satisfaction: A review of issues and concepts. Soc Sci Med.

[2] Howie JG, Heaney DJ, Maxwell M, Walker JJ (1998). A comparison of a Patient Enablement Instrument (PEI) against two established satisfaction scales as an outcome measure of primary care consultation. Fam Pract.

[3] Hudon C, Fortin M, Rossignol F, Bernier S, Poitras M (2011). The Patient Enablement Instrument-French version in a family practice setting: a reliability study. BMC Fam Pract.

[4] Lam C, Yuen N, Mercer S, Wong W (2010). A pilot study on the validity and reliability of the Patient Enablement Instrument (PEI) in a Chinese population. Fam Pract.

[5] Pawlikowska T, Nowak P, Szumilo-Grzesik W, Walker J (2002). Primary care reform: a pilot study to test the evaluative potential of the Patient Enablement Instrument in Poland. Fam Pract.

[6] Pawlikowska T, Walker J, Nowak P, Szumilo-Grzesik W (2009). Patient involvement in assessing consultation quality: a quantitative study of the Patient Enablement Instrument in Poland. Health Expect.

[7] Mead N, Bower P, Roland M (2008). Factors associated with enablement in general practice: cross-sectional study using routine collected data. Br J Gen Pract.

[8] Mercer SW, Jani BD, Maxwell M, Wong S, Watt G (2012). Patient enablement requires physician empathy: a cross-sectional study of general practice consultations in areas of high and low socioeconomic deprivation in Scotland. BMC Fam Pract.

[9] Denley J, Rao J, Stewart A (2003). How do patients rate the quality of consultations in primary care? A patient enablement survey from practices within a primary care trust in Sandwell. Qual Prim Care.

[10] Mead N, Bower P, Hann M (2002). The impact of general practitioners’ patient-centredness on patients’ post-consultation satisfaction and enablement. Soc Sci Med.

[11] Little P, Everitt H, Williamson I, Warner G, Moore M, Gould C (2001). Observational study of the effect of patient centredness and positive approach on outcomes of general practice consultations. BMJ.

[12] Mercer SW, Howie JG (2006). CQI-2 – a new measure of holistic interpersonal care in primary care consultations. Br J Gen Pract.

[13] Howie JG, Heaney DJ, Maxwell M, Freeman GK, Mercer SW (2008). Further observations on enablement. Br J Gen Pract.

[14] Bland JM, Altman DG (1997). Statistics notes. Cronbach’s alpha. BMJ.

[15] Pawlikowska T, Zhang W, Griffiths F, van Dalen J, van der Vleuten C (2012). Verbal and non-verbal behaviour of doctors and patients in primary care consultations – How this relates to patient enablement. Patient Educ Couns.

[16] Haughney J, Cotton P, Rosen JP, Morrison K, Price D (2007). The use of a modification of the Patient Enablement Instrument in Asthma. J Prim Care Respir.

