# Supplementary material for: Reliability and applicability of the Patient Enablement Instrument (PEI) in a Swedish general practice setting
Source: BMC Fam Pract. 2015 Mar 4;16:31. doi: 10.1186/s12875-015-0242-9 (PMC4353449; doi:10.1186/s12875-015-0242-9)
Supplement: Additional file 1: — Swedish version of the PEI adapted from the original English version. [file 12875_2015_242_MOESM1_ESM.pdf]

Appendix 1: Swedish translation of the PEI adapted from the original English version.

Som en följd av ditt läkarbesök känner du att du...

(As a result of your visit to the doctor today, do you feel you are...)

	Mycket bättre *(Much better)*	Bättre *(Better)*	Som tidigare eller sämre *(Same or less)*	Ej relevant *(Not applicable)*
...kan hantera livet *(...able to cope with life)*	☐	☐	☐	☐
...kan förstå din sjukdom *(...able to understand your illness)*	☐	☐	☐	☐
...kan hantera din sjukdom *(...able to cope with your illness)*	☐	☐	☐	☐
...kan hålla dig själv frisk *(...able to keep yourself healthy)*	☐	☐	☐	☐

	Mycket mer *(much more)*	Mer *(more)*	Samma eller mindre *(same or less)*	Ej relevant *(not applicable)*
...är trygg med din hälsa *(...confident about your health)*	☐	☐	☐	☐
...kan hjälpa dig själv *(...able to help yourself)*	☐	☐	☐	☐
